# Multidimensional Chromatographic Fingerprinting Combined with Chemometrics for the Identification of Regulated Plants in Suspicious Plant Food Supplements

**DOI:** 10.3390/molecules28083632

**Published:** 2023-04-21

**Authors:** Surbhi Ranjan, Erwin Adams, Eric Deconinck

**Affiliations:** 1 Section of Medicines and Health Products, Sciensano, J. Wytsmanstraat 14, B-1050 Brussels, Belgium; surbhi.ranjan@sciensano.be; 2 Department of Pharmaceutical & Pharmacological Sciences, Pharmaceutical Analysis, KU Leuven, University of Leuven, Herestraat 49, B-3000 Leuven, Belgium; erwin.adams@kuleuven.be

**Keywords:** plant food supplements, multidimensional fingerprinting, chemometrics

## Abstract

The popularity of plant food supplements has seen explosive growth all over the world, making them susceptible to adulteration and fraud. This necessitates a screening approach for the detection of regulated plants in plant food supplements, which are usually composed of complex plant mixtures, thus making the approach not so straightforward. This paper aims to tackle this problem by developing a multidimensional chromatographic fingerprinting method aided by chemometrics. To render more specificity to the chromatogram, a multidimensional fingerprint (absorbance × wavelength × retention time) was considered. This was achieved by selecting several wavelengths through a correlation analysis. The data were recorded using ultra-high-performance liquid chromatography (UHPLC) coupled with diode array detection (DAD). Chemometric modelling was performed by partial least squares–discriminant analysis (PLS-DA) through (a) binary modelling and (b) multiclass modelling. The correct classification rates (ccr%) by cross-validation, modelling, and external test set validation were satisfactory for both approaches, but upon further comparison, binary models were preferred. As a proof of concept, the models were applied to twelve samples for the detection of four regulated plants. Overall, it was revealed that the combination of multidimensional fingerprinting data with chemometrics was feasible for the identification of regulated plants in complex botanical matrices.

## 1. Introduction

The use of plant food supplements has been established in their popularity in developing countries since time immemorial and is rapidly emerging in the Western world over the past few decades. This tremendous increase in their acceptance can be associated with the consumer perception that everything natural is safe [1,2,3,4]. This is well catalyzed by extensive marketing campaigns, the possibility of self-medication, the ease of purchase without a prescription, and their availability through various sources [5,6,7].

With this rising consumer demand, a huge commercial market emerged, rendering these plant food supplements vulnerable to fraudulent practices. This was propagated by the availability of these products through unregulated sources, particularly ‘the internet’. This put the consumer at high risk [8,9,10,11,12], raising an apprehension about the quality of the available supplements on the market and necessitating their regulation and authentication.

In the European Union (EU), plant food supplements are regulated by the European Directive 2002/46/EC, which allows the commercial sale of plant food supplements after authentication from the member nations and declaration of safety standards [13]. In Belgium, the list of plants and plant parts that are allowed to be used in plant food supplements is regulated by the Royal Decree of 1997 (amended in 2021), which consists of three lists comprising (a) a list of forbidden plants and plant parts for use, (b) a list of edible mushrooms, and (c) a list of plants allowed, but mandating notification to the concerned national authority [14].

The screening of plant food supplements poses a problem due to their complex matrices, a result of the pulverization and mixing of different plants. Therefore, classical macroscopic and microscopic techniques are unsuitable for their identification [15]. The World Health Organization (WHO) recognizes chromatographic fingerprinting as an official technique for the identification and quality determination of herbs [16,17]. Chromatographic fingerprints represent complex chemical profiles of analysed samples which can be obtained through spectroscopic, chromatographic or electrophoretic techniques [18]. They are informative profiles, but they may result in incorrect identification when dealing with closely related species. This problem can be solved by combining chromatographic fingerprinting with multivariate calibration techniques used in chemometrics [17,19]. 

Chemometrics can be defined as a statistical tool that is utilized for solving chemical problems. In the ever-growing world of analytical instruments, it plays an important role in extracting important information from the enormous amount of generated data [20,21] The combination of chromatography and spectroscopy with chemometrics has been used by many authors for the identification of plants using chemical markers [22,23,24]. However, very few have applied this approach to complex herbal matrices [25]. 

This paper is aimed at developing a fingerprinting approach using ultra-high-performance liquid chromatography (UHPLC–DAD) for the detection of four regulated plants (*Aristolochia fanghi*, *Ilex paraguariensis*, *Hoodia gordonii*, and *Garcinia cambogia*, of which *Aristolochia fanghi* falls into list 1 of the Royal Decree and the other three fall in list 3). These are commonly found in plant food supplements claiming weight loss as an indication [26]. Weight-loss supplements have been found to be one of the most adulterated products, owing not only to their use for the treatment of obesity but also to the societal pressure that promotes ‘slim’ figures [6,27,28,29]. 

In order to detect the herbal adulteration of plant food supplements, a unique multidimensional fingerprinting approach was followed in this paper. It takes into account not only classical chromatograms with one wavelength but the whole information consisting of absorbance × retention time × wavelength recorded by diode array detectors. Self-made triturations (using 10 blank herbal matrices and lactose) were prepared in order to validate the method. The recorded data sets were further subjected to a supervised chemometric approach such as partial least squares–discriminate analysis (PLS-DA) for obtaining models and carrying out the classification of the samples. Before modelling, the obtained data were subjected to peak alignment through correlation-optimized warping (COW) in order to reduce any external (analytical) disturbances. It was further explored if it was beneficial to use pretreatment techniques, such as derivatives, standard normal variate (SNV), and autoscaling. Next, different modelling strategies were tested; after selecting the best one, a proof of concept was performed on a set of twelve real samples, out of which nine claimed slimming and three claimed potency enhancement as indications for use. Potency enhancers were added to check the reliability of the model. However, a note should be made that such supplements are very prone to adulteration, and it would be interesting to see if they are adulterated using plants with slimming potential.

## 2. Results 

### 2.1. Final Chromatographic Conditions

The mobile phase consisted of methanol (organic modifier) and 0.1% formic acid in milli-Q water (aqueous component). The column of choice was a C18 Acquity BEH Shield (2.1 mm × 100 mm, 1.7 µm). Methanol/milli-Q water (50:50, *v*/*v*) was selected as the extraction solvent. The column temperature was set at 35 °C. A compromise method for all four plants was difficult to devise, but one common method for a group of two plants could be developed. For *Aristolochia fanghi* and *Hoodia gordonii*, the gradient was set at 90% aqueous phase, held for 30 s, and then decreased to 50% in 1 min. This was held for 1 min, lowered to 10% in 1.5 min, and held for 30 s. Then, the aqueous phase rose again to 90% during 2.5 min and was held for one minute to re-equilibrate the system. The flow rate was 0.25 mL/min. In the case of *Ilex paraguariensis* and *Garcinia cambogia*, the flow rate was 0.30 mL/min and only the gradient differed. The change from 90% to 50% aqueous occurred in 2.5 min and was held for 1.5 min, while the change from 50% to 10% aqueous was faster, occurring within 30 s. The total run time for both methods was only 8 min.

### 2.2. Correlation Analysis and Choice of Wavelengths

Classically, aromatic compounds are recorded at a wavelength of 254 nm. In order to include more information in the data and provide more specific fingerprints, a correlation analysis between the recorded wavelengths (200–400 nm) was carried out. The whole data cube of the reference standard was imported into Matlab to construct a colour map in order to make the first choice of wavelengths. 

**Figure 1:**
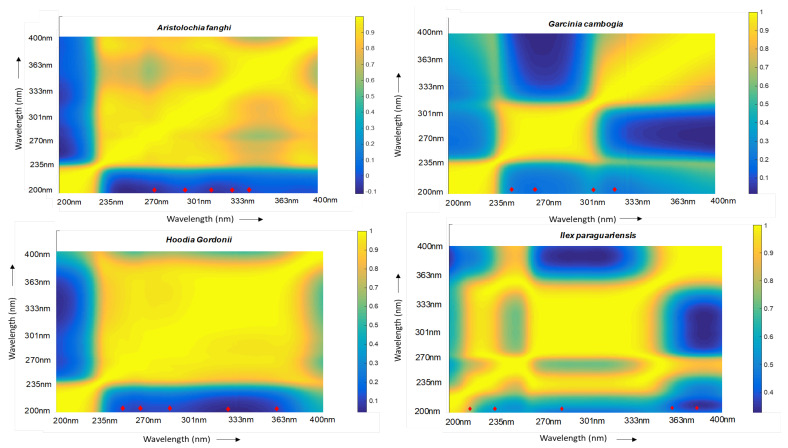
Colour map for *Aristolochia fanghi*, *Garcinia cambogia*, *Hoodia gordonii*, and *Ilex paraguariensis* depicting the correlation between wavelengths. The colour scale represents blue as “highly uncorrelated” and yellow as “highly correlated” wavelengths. The axis forms the wavelengths in nm. The red marker represents an approximation of the selected wavelengths on the colour map for the individual plants. The wavelengths are listed in Table 1.

**Figure 2:**
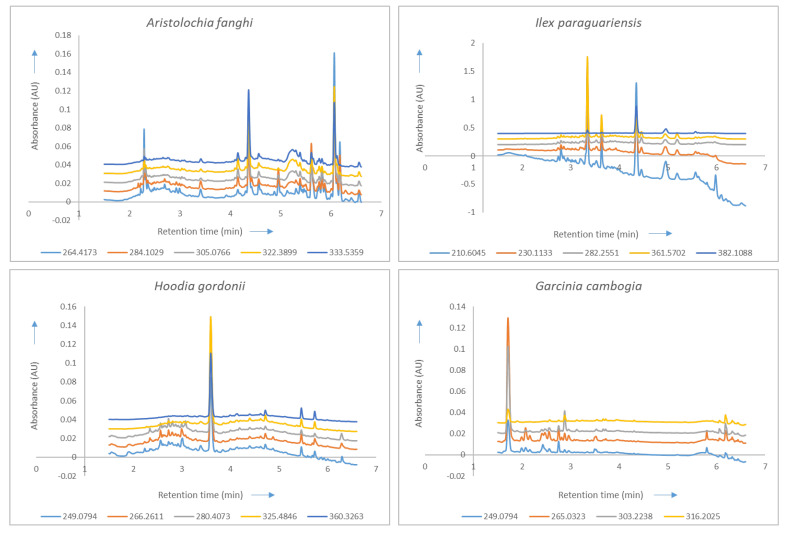
Overlays of the chromatograms of the concerned plants represented at their selected wavelengths (nm) with the *X*-axis representing retention time (in minutes) and the *Y*-axis representing the absorbance (in absorbance units). An offset of 0.01 is added to the chromatograms to provide a better overview of the overlaid wavelengths of each reference plant.

A colour scale was added in order to infer from the map. Areas in ‘blue’ were highly uncorrelated whereas areas in ‘yellow’ (corresponding to a value of 1 from the scale) were highly correlated (Figure 1). Based on the map, the corresponding wavelengths were selected from the data set and correlation coefficients were determined. A selection of a maximum of five wavelengths was made owing to the limitation of calculation power. A cutoff of 0.95 was set and five orthogonal wavelengths were selected for *Aristolochia fanghi*, *Ilex paraguariensis*, and *Hoodia gordonii*, while four were selected for *Garcinia cambogia* (Table 1). An overlay of the chromatograms at each of their respective selected wavelengths is presented in Figure 2.

### 2.3. Chemometric Analysis

A total of 67 samples (55 self-made triturations + 11 blanks + the reference plant), were injected in the UHPLC–DAD system. As already mentioned before, the triturations were composed of 10 herbal matrices (besides lactose) that did not claim slimming as an indication and are not listed as the regulated plants considered in this paper. The raw data of the recorded fingerprints at the selected wavelengths for these triturations were then imported to Excel from Empower. The nature of the data was multidimensional, i.e., consisting of sample × wavelength × retention time as the dimension of the data cube with values constituting absorbance × wavelength × retention time. After selecting the fingerprint region (1.5–6.6 min), the data set was imported into Matlab. Here, 3D matrices were created, which were represented as 67 × 5 × 6121 for binary models and 235 × 5 × 6121 for multiclass models, with 67/235 being the samples, 5 being the wavelengths, and 6121 representing the time points. The data set was then unfolded in order to carry out chemometric treatment owing to the complexity of the data set. The unfolded matrices were represented as 67 × 30,605 and 235 × 30,605. The same representation was obtained for *Garcinia cambogia* but with four wavelengths, and the unfolded matrices were represented as 67 × 24,484 and 235 × 24,484.

In order to avoid any shifts in retention time, the chromatograms for the whole data set as well as real-life samples were aligned using COW. The alignment was performed at all five or four (in the case of *Garcinia cambogia*) wavelengths separately. The target chromatogram for all four plants at each wavelength was then determined by calculating the highest mean. The optimal parametric values of segments and slack were evaluated. A combined data set consisting of all the warped data (at each wavelength) was created for each plant separately. After warping, a separate data set for samples was created and further analysis was performed on the rest. The whole data set was subjected to different data pretreatment techniques. However, no improvement was observed in the results. Therefore, the final data set was considered with just warping as pretreatment.

This data set with triturations and blanks was subjected to the duplex algorithm. After assigning the categorical variables to class 1 as positives for the reference plants and class 2 as negatives (the unspiked or blank matrices), the data for binary modelling were split into two representative sets: a training set (calibration set) and a test set (validation set). For multiclass modelling, five categorical variables were assigned. Each variable represented a different plant and the fifth was blank matrices. More details are represented in Table 2. 

#### 2.3.1. Partial Least Squares–Discriminant Analysis

PLS-DA was carried out to determine whether the samples in the data set could be classified according to the assigned classes. The aim was to create the best model with the least misclassifications in cross-validation and external test set predictions for further testing of real samples. This technique was applied in two approaches of modelling: (a) binary models and (b) multiclass models.

##### Binary Models

The first data set to test this approach was chosen to be that of individual plants consisting of 67 samples in total, with 11 negatives and one reference. Classes were then assigned to positive samples as ‘1′ and negative samples as ‘2′. After defining training and test sets, cross-validation was carried out. A 10-fold cross-validation was used with 30 latent variables to be predicted. After the selection of the most suitable number of PLS factors, modelling was performed. The models were validated using the external test set of 17 samples. 

For *Aristolochia fanghi*, 23 PLS factors gave the best results. A cross-validation of 94% correct classification rate (ccr) was obtained with 47 out of 50 samples in the training set predicted correctly. The three misclassifications were false positives. Modelling results were also acceptable with a ccr of 96%. In order to test the model, external test set validation was performed. The results indicated that only one sample from the test set was misclassified as false positive, and a total ccr of 94% (Table 3) was obtained.

Similarly for *Ilex paraguariensis*, after obtaining the results of cross-validation, 14 PLS factors were selected, giving a ccr of 96% with 48 out of 50 samples predicted correctly. The two misclassified samples were false positives. Modelling results revealed a 100% correct model based on the training set. External test set validation revealed that all samples were predicted correctly except one (Table 3). The misclassified sample here is considered to be a false positive.

For *Hoodia gordonii*, the best cross-validation results were obtained with 20 PLS factors. For the training set, 44 samples were predicted correctly with 6 misclassifications attributed as false positives. Next, modelling was carried out, and a ccr of 96% for the model was determined. Only one misclassified sample was observed for *Hoodia gordonii* after the external test set validation, which was revealed to be a false positive (Table 3). 

For *Garcinia cambogia*, cross-validation of 96% was obtained for 22 PLS factors with the incorrect prediction of only 1 sample out of 50, which was classified as a false positive. The data were then modelled using 22 PLS factors, revealing a ccr of 98%. This model was further validated using the test set. Two samples were misclassified, yielding a ccr of 88% (Table 3). Both samples were revealed to be false positives. 

It is interesting to note that all misclassified samples, apart from two (one in the training set of *Aristolochia fanghi* and the other in the test set of *Ilex paraguariensis*) were all blank or unspiked matrices. A possible explanation could be the variation in the matrix rendering the inability to differentiate between spiked matrices (triturations) and unspiked matrices.

A confusion matrix summarizing the prediction and classification results is depicted in Table 4.

##### Multiclass Models

A cumulative approach to classifying all the plants was attempted by assigning different classes to different plants when developing the model. The modelling was carried out four times, each time using the selected wavelengths for the respective plants (see Section 2.2). For example, a data set using the wavelengths for *Aristolochia fanghi* consisted of samples (triturations of four plants + blanks) at wavelengths of 264 nm, 284 nm, 305 nm, 322 nm, and 333 nm (Table 1); five classes were assigned to each plant and the blank. The data set was composed of 235 samples. The duplex algorithm was performed on this data set, and a total of 59 samples were selected for the test set (Table 2). A similar approach to binary modelling was followed.

Multiclass modelling for data consisting of wavelengths for *Aristolochia fanghi* revealed a ccr for cross-validation equal to 91% using 22 PLS factors with 161 out of 176 samples in the training set predicted correctly. During cross-validation, six false positives for *Aristolochia Fanghi* were observed, from which, respectively, three, one, and two samples were triturations of *Ilex paraguariensis, Garcinia cambogia*, and the blanks. Similarly, one trituration of *Ilex paraguariensis* and five blanks were misclassified as *Hoodia gordonii*. *Ilex paraguariensis* was falsely detected in two samples of *Garcinia cambogia*, and *Garcinia cambogia* in one sample of *Hoodia gordonii.* Modelling was carried out to obtain a ccr of 93%, and the external test set validation resulted in six misclassifications with a ccr of 90%. When evaluated, the misclassifications were found for *Hoodia gordonii*, which was falsely classified as positive in three blank samples and one sample of *Ilex paraguariensis*, whereas one sample of *Ilex paraguariensis* and one blank sample were misclassified as containing *Garcinia cambogia* (Table 5 and Table 6).

For the model with wavelengths of *Ilex paraguariensis*, 18 PLS factors were chosen, and a ccr of 93% was obtained by cross-validation, revealing the correct prediction of 164 samples in the training set. *Ilex paraguariensis* was further detected as a false positive for the blanks, whereas *Garcinia cambogia* was found to be positive for one sample in *Hoodia gordonii* and four blank samples. Moreover, *Aristolochia fanghi* was found to be present in one sample of *Hoodia gordonii*, whereas *Hoodia gordonii* was found to be falsely positive in samples of *Garcinia cambogia* and blanks with values of three and two, respectively. Modelling results showed sufficiently good models with a ccr of 97%. Seven misclassifications were observed when validation with an external test set was carried out. Out of these seven misclassifications, one and three false positives for *Aristolochia fanghi* were detected in *Hoodia gordonii* and the blanks, respectively. *Hoodia gordonii* was found to be positive in one trituration of each *Garcinia cambogia* and the blank sample, and one sample of *Ilex paraguariensis* was misclassified as blank (Table 5 and Table 6).

Similarly, for the model constructed with wavelengths of *Hoodia gordonii*, the best modelling results were obtained with 23 PLS factors, and a ccr of 87% was obtained by cross-validation. One hundred fifty-three of the training set samples were predicted correctly. *Hoodia gordonii* was found to be positive in five samples of *Ilex paraguariensis*, in one sample of *Garcinia cambogia*, and in four blank samples. *Aristolochia fanghi* was misclassified as falsely positive, amounting to one trituration of *Hoodia gordonii*, one of *Ilex paraguariensis*, four of *Garcinia cambogia*, and two of the blank samples. On the other hand, for *Ilex paraguariensis*, one false positive was evaluated in the blank sample, and for *Garcinia cambogia*, a total of four misclassifications (all false positives) were found as one in *Hoodia gordonii* and three in *Ilex paraguariensis*. The ccr of modelling was found to be 94%. The validation results using the test set revealed six misclassifications with 90% ccr, all of which were false positives of *Hoodia gordonii* in triturations of *Garcinia cambogia* (1) and the blanks (5) (Table 5 and Table 6).

When modelled with 22 PLS factors, the data set with the wavelengths of *Garcinia cambogia* showed a ccr of 84% by cross-validation with 28 misclassifications for the training set. It was revealed that *Garcinia cambogia* was predicted as falsely positive in one trituration of *Ilex paraguariensis*, three of *Aristolochia fanghi*, six of *Hoodia gordonii*, and three of the blanks. *Ilex paraguariensis* was also predicted as a false positive in *Hoodia gordonii*. Meanwhile, for *Aristolochia fanghi*, two false positives were observed in *Garcinia cambogia*, two in *Ilex paraguariensis*, five in *Hoodia gordonii*, and one in the blanks. For *Hoodia gordonii*, *Garcinia cambogia* consisted of two misclassified predictions, and two samples of the blanks were falsely predicted as *Garcinia cambogia*. External test set validation results comprised six misclassifications with a ccr of 90%. *Garcinia cambogia*, *Aristolochia fanghi*, and *Hoodia gordonii* were all classified as falsely positive, respectively, in one, two, and two blank samples, whereas one blank was misclassified as positive in triturations of *Ilex paraguariensis* (Table 5 and Table 6). 

A common pattern in multiclass models was the higher amount of misclassifications when compared with binary models. It was observed that blank matrices were not predicted correctly. A reason could be that the features of the blank class were not strong enough to differentiate between spiked and unspiked mixtures, which is contrary to what was seen in binary modelling, where a maximum of two misclassifications was obtained for the external test set validation. 

Furthermore, the best-performing multiclass model was chosen for the prediction of real samples. This was achieved by comparing the ccr% of all the models along with their misclassifications, which led to the choice of model based on the wavelengths of *Aristolochia fanghi* (for reference see Supplementary Material Figures S1 and S2).

#### 2.3.2. Real Samples

As proof of concept, 12 samples that showed slimming (9 samples) and potency enhancement (3 samples) as indications were tested. Out of 12, using binary modelling 10 samples were found to be positive for a regulated plant. Herein, six were detected as positive for *Hoodia gordonii*, four were positive for *Ilex paraguariensis*, and seven were positive for *Garcinia cambogia*. Samples were found to be positive for several plants as summarized in Table 7. Surprisingly, *Aristolochia fanghi* was also found in four samples, even though it is a banned product in Belgium.

For the rest, samples that claimed to be positive for a regulated plant on the label (samples 3 and 7 for *Garcinia cambogia* and sample 6 for *Ilex paraguariensis*) were also found to be positive by our modelling approach.

The multiclass model constructed using wavelengths of *Aristolochia fanghi*, when used for the prediction of the real samples, could detect 11 positives out of 12 samples. Four positives were found for *Aristolochia fanghi*, one for *Ilex paraguariensis*, two for *Hoodia gordonii*, and four for *Garcinia cambogia*. A brief overview of sample prediction by multiclass modelling is represented in Table 7.

When comparing the results of the two approaches, similarities between sample prediction for samples 1, 3, 6, 7, 8, 9, 10, and 11 were found. Whereas a comparative study could depict the presence of multiple plants in the sample for binary modelling, there were no such classifications for multiclass modelling. Therefore, we need to start a discussion about the choice between the most feasible approach for modelling using PLS-DA.

#### 2.3.3. Comparison of Results Obtained for 254 nm and for Selected Wavelengths

The same approach as described above was carried out for binary modelling using two data sets, one comprising data obtained at 254 nm and the other with data obtained for multiple wavelengths. From the results, it was observed that the error in prediction of the external test set was higher for 254 nm for the considered plants when compared with multidimensional data. For example, when comparing the result for *Aristolochia fanghi*, the ccr for external test set prediction at 254 nm was 88%. On the other hand, a ccr of 94% (Table 3) was observed for multidimensional data. For *Hoodia gordonii*, a ccr of 82% was obtained at 254 nm, while 94% (Table 3) was observed for the multidimensional data. Moreover, for both *Ilex paraguariensis* and *Garcinia cambogia*, a ccr% of 76% was observed when considering 254 nm, while ccr’s of 94% and 88%, respectively, were recorded for multidimensional data. Even though the ccr values for modelling are sometimes comparable, the addition of multiple wavelengths adds to the specificity and robustness of the models. Therefore, this approach is superior to the use of just a single wavelength.

## 3. Materials and Methods

### 3.1. Samples and Reagents

Reference material for *Aristolochia fanghi*, *Ilex paraguariensis*, *Hoodia gordonii*, and *Garcinia cambogia* was obtained from the American Herbal Pharmacopoeia (Scotts Valley, CA, USA), which authenticated the plant material through different macro- and microscopic techniques and provided a validation certificate confirming its identity.

Reagents such as methanol (HPLC grade), acetonitrile (HPLC grade), and ethanol (96% *v*/*v*) were obtained from Biosolve (Valkenswaard, The Netherlands). Formic acid (99.7%), hydrochloric acid solution (37 *w*/*w*%), and ammonia solution (25 *w*/*w*%) were all purchased from Merck (Darmstadt, Germany). A Millipore—MilliQ^®^ system (Billerica, MA, USA) was used to dispense milli-Q water. Lactose was also procured from Merck (Darmstadt, Germany).

All botanical supplements used for triturations and samples were selected from the samples seized by the Federal Agency for the Safety of the Food Chain (FASFC) (Brussels, Belgium) and sent to the laboratory to be tested for chemical adulteration. A choice for the botanical matrices to be used for triturations was made such that no concerned plant in this paper was listed on the label, and the supplement itself did not mention slimming as an indication. On the other hand, 12 samples were chosen, out of which 9 (samples 1–9) were slimming aids while 3 (samples 10–12) were potency enhancers.

### 3.2. Sample Preparation

#### 3.2.1. Preparation of Reference Solutions

The dried plant parts were crushed into powder using mortar and pestle and then sieved through a 70 µm pore-sized sieve. For UHPLC, reference solutions (10 mg/mL) were prepared using methanol and milli-Q water (50:50, *v*/*v*) as extraction solvent. After mixing and vortexing for 30 s, they were placed in an ultrasound bath for 40 min. The solutions were then filtered using a 0.2 µm PTFE filter and collected in vials.

#### 3.2.2. Preparation of Triturations

Self-made triturations were prepared for each reference plant using 10 botanical matrices and lactose. The triturations were prepared by mixing the reference plant and the botanical supplement in a classical concentration range from 5% to 50% and 5 concentrations: 1/20, 1/15, 1/10, 1/5, and 1/2 (reference/botanical supplement). Therefore, for each plant, a set of 55 (11 × 5) triturations was obtained. This mixture was then homogenised using a mortar and pestle. The triturations with reference plant material and blanks (unspiked matrices) were injected in the UHPLC-DAD system using the same extraction procedure as the reference standards. The data collected was used for further chemometric processing.

#### 3.2.3. Preparation of Samples

Twelve real samples were used for testing the developed models. All samples were present in capsules, in powdered form. Hence, the procedure to extract and prepare reference solutions was used. Out of the selected samples, samples 3 and 7 claimed the presence of *Garcinia cambogia* and sample 6 claimed the presence of *Ilex paraguariensis.*

### 3.3. Instrumentation and Conditions

#### 3.3.1. Preparation of Reference Solutions

All solutions from standards, triturations, and samples were injected into a UHPLC-DAD system from Waters (Milford, MA, USA). The system consisted of a binary pump, a column oven, and a temperature-regulated autosampler coupled with a DAD detector.

##### Method Development

A previously developed HPLC-DAD method was used as starting point for *Aristolochia fanghi* and *Ilex paraguariensis* [25]. This was transferred to a UHPLC system by adapting and optimizing the chromatographic parameters.

For *Hoodia gordonii* and *Garcinia cambogia*, method development was started by conducting a full factorial experimental design to find the most optimal features. The parameters considered were as follows: type of column, extraction solvent, pH of the aqueous phase, and organic modifier. In order to select the most optimum conditions, a cutoff value of 1000 µV was set for absorbance. Four different reversed-phase columns were tested: C8 Acquity UPLC (2.1 mm × 100 mm, 1.7 µm), C18 Acquity UPLC BEH (2.1 mm × 100 mm, 1.7 µm), C18 Acquity BEH Shield (2.1 mm × 100 mm, 1.7 µm), and HSS T3 Acquity UPLC (2.1 mm × 100 mm, 1.8 µm). The Waters Acquity BEH Shield column (2.1 mm × 100 mm, 1.7 µm) was found to be best suitable. Even though the C18 BEH and HSS T3 showed 13 peaks falling above the cutoff, the BEH Shield yielded many more peaks. Concerning the choice of organic modifier and pH, it was found that methanol in combination with formic acid (0.1%) showed the best response and separation of the peaks. In order to optimise the gradient, different time points were tried. The most optimal gradient was found to be the one described in Section 2.1. After testing different flow rates, 0.25 mL/min was revealed to be the most suitable choice. The column temperature was also investigated and after comparing the chromatograms at 30 °C, 35 °C, and 40 °C, 35 °C produced the best chromatograms with good separation. The repeatability of extraction and injection was further evaluated and good results were obtained for the chromatograms.

Similar results were obtained for *Garcinia cambogia* with differences in the gradient and flow rate (0.30 mL/min). An effort was made to find a compromise method for the four plants. However, it was found that two different chromatographic methods would be better per set of two different plants *(Ilex paraguariensis* and *Garcinia cambogia* vs. *Aristolochia fanghi* and *Hoodia gordonii).*

### 3.4. Selection of Wavelengths

To select orthogonal wavelengths that can provide maximum information for a certain plant, a correlation analysis for the reference plants was performed between wavelengths recorded from the DAD detector. In the first step, the whole data cube was exported from Empower and imported into Matlab. A colour map was created between wavelengths (represented as wavelength number) on both *x*- and *y*-axis. A gradient scale was added to further distinguish between correlated and uncorrelated wavelengths. While blue represented uncorrelated regions, yellow represented correlated regions. The wavelengths falling within the blue region were then selected from the whole data set. Following this, the correlation coefficients were calculated using Excel. Further filtering of wavelengths by setting a cut-off limit of 0.95 between adjacent wavelengths was established. Finally, a choice for the most orthogonal wavelengths was made.

### 3.5. Data

#### 3.5.1. Data Set Preparation and Multidimensional Fingerprints

All recorded DAD data from Empower were exported to Excel to prepare the data sets. A fingerprint region that represented the maximum information (most number of peaks) characterising the plant, was visually selected on the chromatogram. This was found to be from 1.5 to 6.6 min. Peaks occurring before and after this region were not considered for further analysis.

In this paper, the whole data cube recorded on a UHPLC system was taken into account, (i.e., absorbance × wavelength × retention time), thus creating a multidimensional fingerprint. As conventional chromatographic fingerprints at 254 nm lack specificity in cases of herbal mixtures, a multidimensional fingerprint should tackle this problem.

Two strategies for the preparation of the data set were followed. In the first one, a data set containing orthogonal wavelengths based on correlation analysis was prepared. In the second approach, the whole data cube consisting of all the wavelengths was considered. Both data sets were then imported into Matlab. Unfortunately, the size of the whole data cube was too large for Matlab. Therefore, only the data set with orthogonal wavelengths was selected.

#### 3.5.2. Peak Alignment

Dealing with enormous chromatographic data comes with disadvantages. Due to changes between days, such as small differences in mobile phase composition or tiny machine instabilities, shifts in peaks along the time axis may arise. This necessitates alignment since such adjustments are prerequisites prior to further chemometric treatment [30].

The principle of COW is based on sequential stretching and compression along the time axis. The optimal alignment is determined by the correlation coefficient between the reference and the sample chromatogram [31]. The first step in this technique is to find a target profile (T) in the chromatograms. This is carried out by calculating the highest mean of correlation coefficients between the chromatograms. The sample chromatogram is aligned using the target as a reference. Next, the sample chromatogram is to be aligned and the reference chromatogram is divided into subsections. Each subsection (i) of the sample is then compared with the *i*th section of the reference and warped accordingly. By changing the position of the endpoint by a finite number of lengths, called slack parameters (s), a segment can be stretched or compressed. The stretching and compression of the sample chromatogram are linearly interpolated on the reference chromatogram. This process is then continued with the endpoint of the previous section becoming the starting point of the next section. The quality of the warped chromatograms is assessed by the calculation of correlation coefficients between the section of the sample chromatogram and its corresponding section on the target profile. Only the ideal combinations of warping are kept while the suboptimal ones are discarded during dynamic programming. This ideal combination constitutes the highest cumulative value of the correlation coefficients [30,32].

The data set with triturations was combined with those consisting of samples and blanks, followed by COW. The segment length was varied between 10 and 50, whereas 1–5 slack parameters were tested for each segment. After warping, the data sets were split again into their normal arrangements.

#### 3.5.3. Data Pretreatment

Data pretreatment was carried out using various preprocessing techniques that included autoscaling, snv, derivatives (1st and 2nd), and combination of different preprocessing techniques, for example, autoscaling with derivatives. However, when comparing treated data with untreated data, the modelling scores using PLS-DA were not significantly different. Therefore, no pretreatment technique (after warping) was carried out.

#### 3.5.4. Test Set Selection

The duplex algorithm was used for splitting the data set into representative sets, involving a calibration and validation set, independent of each other. It started by choosing two points that were farthest away by calculating the Euclidean distance between them and placing them in the calibration set. Subsequently, the pair that was the second furthest away was selected and added to the validation set. This alternation between grouping into calibration and validation set continued until all the points were appointed. An optimal validation set is one that contains about 20% of the samples from its total data set [33].

In this study, 25% of the data set was selected for the test set (validation set).

#### 3.5.5. PLS-DA

PLS-DA is a supervised technique that is used to discriminate and classify different samples. It is a dimension reduction technique that is an extension of PLS regression when the response variable (y) is categorical. The technique defines PLS factors that are linear combinations of original variables [34]. Cross-validation is very important when performing PLS-DA as they are prone to overfitting [35]. Tenfold cross-validation was done and PLS factors showing maximum ccr% were selected for modelling. After modelling, external test set validation was carried out to test the predictive ability of the model. The misclassifications and ccr% were comprehended and compared between different models of different plants or different approaches. The two different approaches applied here were binary modelling and multiclass modelling.

#### 3.5.6. Software

Empower 3 software from Waters (Milford, MA, USA) as used for data acquisition from UHPLC-DAD. Matlab version 2016b from Mathworks (Natick, MA, USA) was used for data processing and modelling. The ChemoAc toolbox, version 4 (Brussels, Belgium) was used for the application of PLS-DA.

## 4. Discussion and Conclusions

In this paper, a feasibility study was carried out to combine the use of multidimensional fingerprints with chemometric modelling. Classically, chromatographic fingerprinting is used for differentiation using visual inspection or similarity analysis. However, these techniques fall short when complex herbal matrices are considered.

Starting with the development of the chromatographic method, there were attempts to find a compromise in order to analyse the plants simultaneously using one method. However, two separate methods for the four plants were needed. The selection of wavelengths was carried out using correlation analysis. Five wavelengths were selected for three plants; four, for *Garcinia cambogia.* The addition of more wavelengths in the data set rendered the chromatogram more specific, which is of key importance when comparing different fingerprints. This can also be confirmed when comparing the chromatograms for all the plants at 254 nm and then comparing them to those occurring at the corresponding wavelengths. All data were imported into Matlab for modelling with PLS-DA.

In this study, two modelling approaches were carried out. The binary models demonstrated very good predictive abilities for all four plants. A maximum of two misclassifications were observed for the external test set of *Garcinia cambogia.* From these results, one could conclude that PLS-DA was able to model the discrimination between plants in line with the aim of this paper.

The multiclass models, surprisingly, showed acceptable results, but with higher misclassifications. In addition, another pattern was seen for the results since the negative class 5 could not be predicted during the external test set validation.

In a common pattern observed for both binary models and multiclass models, a high number of PLS factors were chosen for modelling. This can be explained by taking into consideration that the data set had high complexity along with considerable variations. The optimal choice of PLS factors was carried out by reviewing the ccr% obtained through cross-validation (for reference see Supplementary Material Figures S1 and S2).

As proof of concept, a small selection of samples was tested using the developed models. This sample set consisted of nine samples sold as slimming aids and three as potency enhancers. The testing of binary models revealed that all samples that claimed the presence of a certain plant on the packaging were found positive for the concerned plant, i.e., two for *Garcinia cambogia* and one for *Ilex paraguariensis*. In general, the popularity of *Ilex paraguariensis*, *Hoodia gordonii*, and *Garcinia cambogia* is logical as their circulation in the market increased considerably, especially for the latter two [26]. It was surprising that *Aristolochia fanghi* was detected in the four plant samples. According to the Royal Decree of 1997, it is banned as a food supplement in Belgium. Though these results should be interpreted with caution, in this study we dealt with unnotified samples, i.e., originating from the internet market. If *Aristolochia fanghi* would be detected in a notified product, which is legally present on the market, the confirmatory analysis would be necessary using, e.g., high-resolution mass spectrometry or DNA-based methods, such as PCR.

Another observation made from the results was that multiple plants could be detected in single samples, even though no claims for them were made on the label, thus making it important to alert the national authorities. An additional consideration was made regarding the three potency enhancers. Each sample tested positive for at least one of the investigated plants. This indicates that there is a possibility that potency enhancers can be contaminated or adulterated with slimming plants in order to provide two benefits through one ‘magic bullet’.

On the other hand, sample prediction through multiclass models revealed a few concerns with regard to the technique itself. Instead of predicting all present plants considered in this paper in the samples, the predictive ability might be restricted to the one having higher absorbance. Another shortcoming is that when new plants need to be analysed using this model, all the plants should be reinjected, which is not practical. Thus, these models should be constantly updated. So, binary models are preferred over multiclass models.

Overall, it can be concluded that chromatographic fingerprinting in combination with chemometrics can be used to classify regulated plants for their presence or absence in complex plant matrices of suspected herbal supplements with a high degree of correctness. The use of multiple wavelengths also shows advantages towards specificity and model robustness compared with the use of single-wavelength fingerprints, especially when dealing with complex herbal mixtures or matrices.

Based on the proof of concept using 12 real samples, it can be concluded that herbal adulteration is a problem, especially with samples purchased from irregular sources, such as uncontrolled websites, and may constitute a threat to consumer health.

## Figures and Tables

**Table 1:** Selected wavelengths (nm) for all 4 plants.

*Aristolochia fanghi*	*Ilex paraguariensis*	*Hoodia gordonii*	*Garcinia cambogia*
264	210	249	249
284	230	266	265
305	282	280	303
322	361	325	316
333	382	360	

**Table 2:** Overview of results obtained from duplex showing the attribution of samples (objects) into the training set and test set.

Type of Data Set	Class Assigned	Total Samples in the Data Set	No. of Samples in Training Set	No. of Samples in Test Set
Binary data set	1, 2	67	50	17
Multiclass data set	1, 2, 3, 4, 5	235	176	59

**Table 3:** Binary modelling using PLS-DA with 67 total samples.

Slimming Plant	PLS Factors	Cross Validation (ccr%) with Misclassified Samples between Brackets ()	Modelling (ccr%)	External Test Set Prediction (ccr%) with Misclassified Samples between Brackets ()
*Aristolochia fanghi*	23	94% (3/50)	96%	94% (1/17)
*Ilex paraguariensis*	14	96% (2/50)	100%	94% (1/17)
*Hoodia gordonii*	20	88% (6/50)	96%	94% (1/17)
*Garcinia cambogia*	22	96% (1/50)	98%	88% (2/17)

**Table 4:** The overall depiction of the confusion matrix summarizing best predicting binary models for the 4 plants. The training set consists of 50 samples and the test set consists of 17 samples.

Slimming Plant	True Positives Training Set Test Set (cv)		False Positives Training Set Test Set (cv)		True Negatives Training Set Test Set (cv)		False Negatives Training Set Test Set (cv)	
*Aristolochia fanghi*	45	12	2	1	3	4	0	0
*Ilex paraguariensis*	43	12	2	0	5	4	0	1
*Hoodia gordonii*	42	14	6	1	2	2	0	0
*Garcinia cambogia*	43	13	2	2	5	2	0	0

**Table 5:** Multiclass modelling by PLS-DA depicting the best models selected for each plant. The total data set consisted of 235 samples.

Slimming Plant	PLS Factors	Cross Validation (ccr%) with Misclassified Samples between Brackets ()	Modelling (ccr%)	External Test Set Validation (ccr%) with Misclassified Samples between Brackets ()
*Aristolochia fanghi*	22	91% (15/176)	93%	90% (6/59)
*Ilex paraguariensis*	18	93% (12/176)	97%	88% (7/59)
*Hoodia gordonii*	23	87% (23/176)	94%	90% (6/59)
*Garcinia cambogia*	22	84% (28/176)	93%	90% (6/59)

**Table 6:** The overall depiction of the confusion matrix for multiclass modelling for 4 separate models constructed using the wavelength of that plant. The total sample set comprised 235 samples, and each training and test set formed 176 and 59 samples, respectively.

Slimming Plant	True Positives Training Set Test Set (cv)		False Positives Training Set Test Set (cv)	
A multiclass model with wavelengths of *Aristolochia fanghi*				
*Aristolochia fanghi*	43	13	6	0
*Hoodia gordonii*	49	6	6	4
*Ilex paraguariensis*	29	20	2	0
*Garcinia cambogia*	39	14	1	2
Blank	0	0	0	0
A multiclass model with wavelengths of *Ilex paraguariensis*				
*Aristolochia fanghi*	46	10	1	4
*Hoodia gordonii*	44	9	5	2
*Ilex paraguariensis*	34	21	1	0
*Garcinia cambogia*	40	12	5	0
Blank	0	0	0	1
A multiclass model with wavelengths of *Hoodia gordonii*				
*Aristolochia fanghi*	43	11	8	0
*Hoodia gordonii*	46	10	10	6
*Ilex paraguariensis*	26	23	1	0
*Garcinia cambogia*	38	8	4	0
Blank	0	1	0	0
A multiclass model with wavelengths of *Garcinia cambogia*				
*Aristolochia fanghi*	40	12	10	2
*Hoodia gordonii*	35	10	2	2
*Ilex paraguariensis*	27	23	1	0
*Garcinia cambogia*	45	8	13	1
Blank	0	0	2	1

**Table 7:** Depiction of obtained predicted samples by comparing binary modelling for the four plants and multiclass model based on the selected wavelengths for *Aristolochia fanghi*.

Samples	Plants Classified According to Binary Modelling (Comparative)	Plants Classified with the Multiclass Model
Sample 1	*Ilex paraguariensis*, *Garcinia cambogia*	*Garcinia cambogia*
Sample 2	--	*Garcinia cambogia*
Sample 3	*Aristolochia fanghi*, *Hoodia gordonii*, *Garcinia cambogia*	*Hoodia gordonii*
Sample 4	*Hoodia gordonii*, *Ilex paraguariensis*	*Aristolochia fanghi*
Sample 5	*Hoodia gordonii*,	*Aristolochia fanghi*
Sample 6	*Garcinia cambogia*, *Ilex paraguariensis*	*Ilex paraguariensis*
Sample 7	*Aristolochia fanghi*, *Hoodia gordonii*, *Garcinia cambogia*	*Hoodia gordonii*
Sample 8	--	--
Sample 9	*Aristolochia fanghi*, *Hoodia gordonii*, *Garcinia cambogia*	*Garcinia cambogia*
Sample 10	*Garcinia cambogia*, *Ilex paraguariensis*	*Garcinia cambogia*
Sample 11	*Aristolochia fanghi*, *Hoodia gordonii*	*Aristolochia fanghi*
Sample 12	*Garcinia cambogia*	*Aristolochia fanghi*

## Data Availability

Not applicable.

## Supplementary Materials

The following supporting information can be downloaded at: https://www.mdpi.com/article/10.3390/molecules28083632/s1, Figure S1: Ccr% vs. number of PLS factors (binary modelling); Figure S2: Ccr% vs. number of PLS factors (multiclass modelling).

## Sample Availability

The samples were provided by the Federal Agency for the Safety of the Food Chain (FASFC), Brussels, Belgium.
