# Supplementary material for: Multidimensional Chromatographic Fingerprinting Combined with Chemometrics for the Identification of Regulated Plants in Suspicious Plant Food Supplements
Source: Molecules. 2023 Apr 21;28(8):3632. doi: 10.3390/molecules28083632 (PMC10146433; doi:10.3390/molecules28083632)
Supplement: Supplementary file 1 [file molecules-28-03632-s001.zip › molecules-2306112-supplementary.pdf]

Supplementary material

Title: Ccr% vs number of PLS factors (binary modelling)

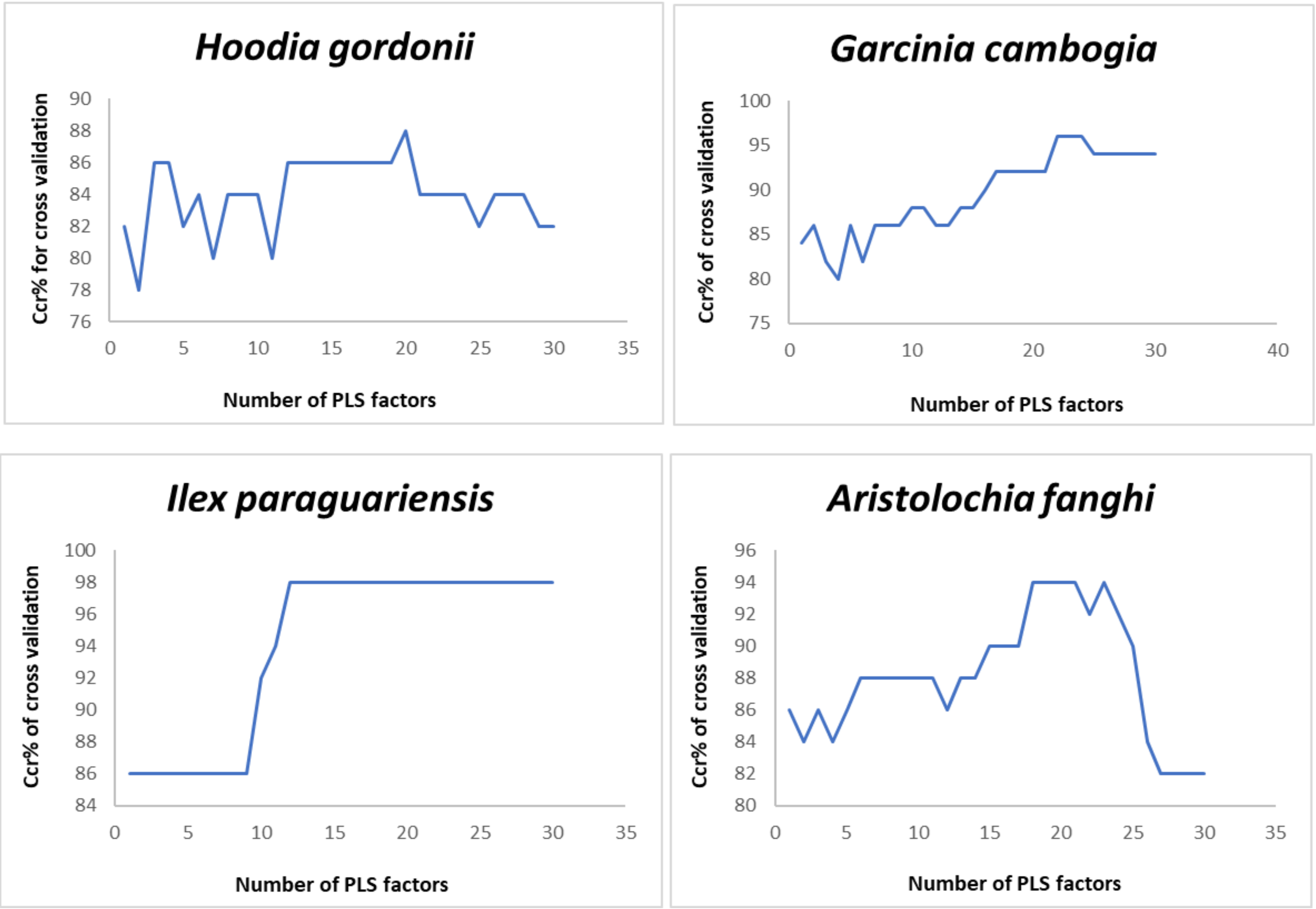

Figure S1. A correct classification rate% vs the number of PLS factors has been represented in the graphs above. As interpreted from the graph high PLS factor was selected based on the ccr% and taking into consideration the external test set error of prediction. This representation is done for binary models of each individual plants.

Title: Ccr% vs number of PLS factors (Multiclass modelling modelling)

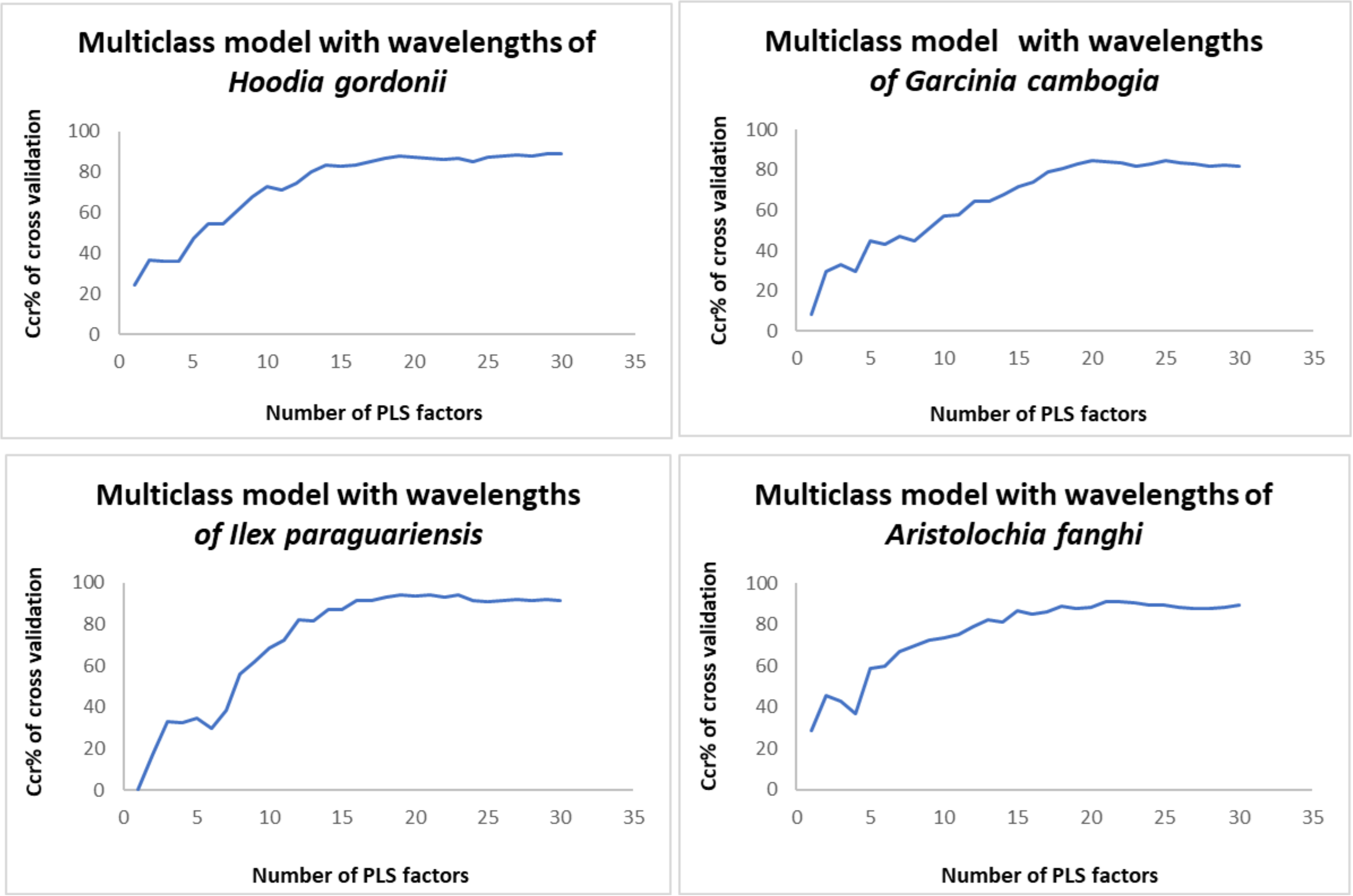

Figure S2. A correct classification rate% vs the number of PLS factors has been represented in the graphs above. As interpreted from the graph high PLS factor was selected based on the ccr% and taking into consideration the external test set error of prediction. This representation is done for multiclass models with wavelengths of each plant respectively.
